# Psychomotor function measured via online activity predicts motor vehicle fatality risk

**DOI:** 10.1038/s41746-017-0003-3

**Published:** 2018-01-15

**Authors:** Tim Althoff, Eric Horvitz, Ryen W. White

**Affiliations:** 1 Stanford University, Stanford, CA USA; 2 Microsoft, One Microsoft Way, Redmond, WA 98052 USA

**Keywords:** Epidemiology, Risk factors, Fatigue, Epidemiology

## Abstract

Impaired psychomotor performance severely increases the risk of fatal and non-fatal car accidents. However, we currently lack methods to continuously and non-intrusively monitor psychomotor performance. We show we can estimate psychomotor function at population scale from 16 billion observations of typing speeds during the input of web search queries. We show that these estimates exhibit diurnal variation with a substantial increase during typical sleep times, matching published accident risk rates. Further, we show that psychomotor impairment, as measured by keystroke timing, predicts motor vehicle fatality risk on a population level (Spearman *ρ* = 0.61; *p* « 10^−10^). The methods and results highlight a promising direction of harnessing ambient streams of data, such as patterns of interactions with devices, as large-scale sensors to continuously and non-intrusively monitor human psychomotor performance at population scale.

## Introduction

Motor vehicle crashes are responsible for 1.25 million deaths annually and are the leading cause of death for people of ages 15–29.^1^ The risk of car crashes based on operator error increases significantly with insufficient sleep.^2,3^ Recent advances in inferring psychomotor function using measures of typing speed of queries during web search enable population-scale estimation of psychomotor impairment.^4^ We examine whether psychomotor performance as indicated by slower typing speeds during web search predicts population-level motor vehicle fatalities by locale.

## Results

**Fig. 1:**
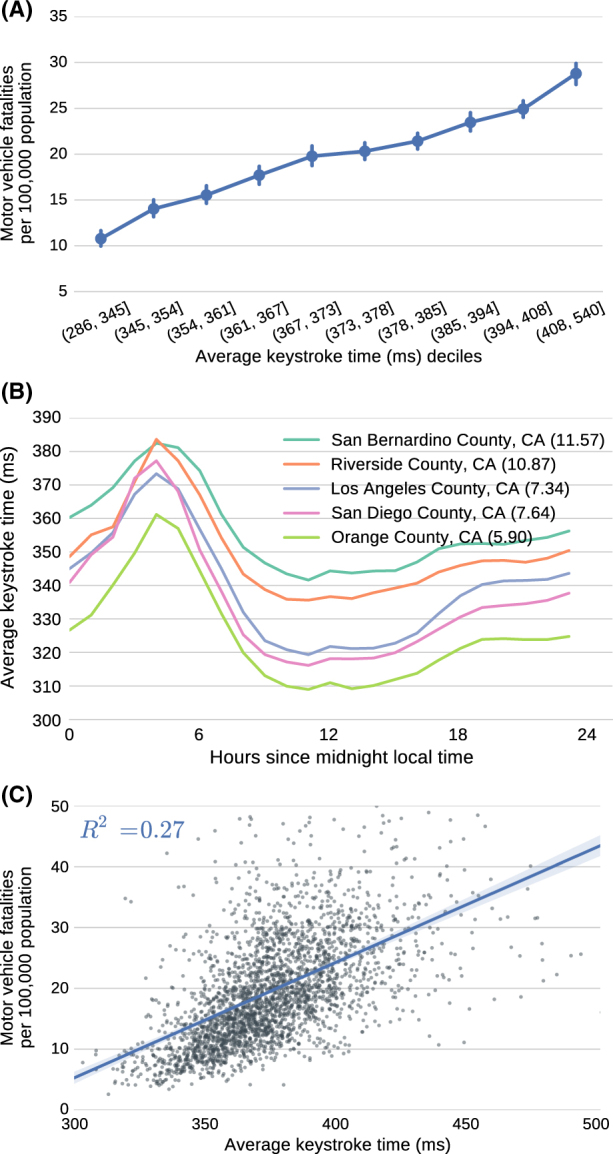
**a** Keystroke timing is strongly correlated with the population-normalized number of motor vehicle fatalities across 2723 US counties. **b** Average keystroke timing over time for five example counties in California. The number in parentheses after each county name refers to the population-normalized number of motor vehicle fatalities. **c** Number of motor vehicle fatalities (population-normalized) by average keystroke timing across 2723 US counties

County-level average keystroke timing is strongly correlated with motor vehicle fatalities across 2723 counties (Spearman *ρ* = 0.61; *p* « 10^−10^; Fig. 1a). Controlling for potential confounding factors in a multivariate linear regression, keystroke timing remains a statistically significant predictor of motor vehicle fatalities (*p* « 10^−10^; *t*-test; Adj. *R*^2^ = 0.554; *N* = 2,555). This correlation is further illustrated through the ordering by keystroke timing for the five largest California counties by population (Fig. 1b). For example, San Bernadino County has the highest average keystroke times and also the largest number of deaths due to traffic accidents (11.57 per population of 100,000). Additionally, the diurnal variation in keystroke timing matches that of published accident risk rates with a substantial increase during typical sleep times.^5^

## Discussion

Interactions with a web search engine enable inferences about accident risk on a national scale. The study was limited by its cross-sectional and correlational design. Search queries and accident risk data were collected during different time periods and included subjects do not necessarily overlap. However, fatality rates have been highly correlated from year to year (Supplementary Figs. 1 and 2), suggesting that our statistical analyses are insensitive to the shift in time of the observational window.

Beyond uses in population-level studies, patterns of interaction with online services may enable continuous, longitudinal monitoring of individuals for fatigue and other forms of psychomotor impairment. Future research directions include investigation of the predictive power of keystroke timing on accident risk on an individual level.

## Methods

Centers for Disease Control and Prevention mortality statistics^6^ from 2007 to 2013 (the most recent year available) were used to compute population-normalized, annual mean motor vehicle mortality rates on a county level. Year-to-year fatality rates have been stable over time within individual counties (Supplementary Figs. 1 and 2).

Average keystroke timings across all US counties over a 4-month interval (April–July 2016) were computed from archival, de-identified search query logs from the Bing web search engine of data routinely collected for improving search results and permitted through Bing’s Terms of Service. Only queries from desktop and notebook computers were used and queries from mobile devices were excluded. Keystroke timing is defined as the time in milliseconds between two key-down events and is estimated from consecutive search engine requests as detailed in Althoff et al.^4^ Search engine requests from counties with less than 10,000 keystrokes in total (4.7%) were excluded. The sample includes 16.1 billion timed keystrokes over 2723 counties. Keystroke timing estimates have high precision due to the large number of keystrokes from each individual country (e.g., 393 million keystrokes from Los Angeles county alone).

To control for potential confounding, a multivariate linear regression analysis was performed that included the following factors^6^ (using all 2555 counties with complete records out of 2723 total counties): age (fraction of population below 18 or above 65), gender (% female), education (% graduated from high school, % with some college education), income (median household income), unemployment (% civilian labor force that is unemployed and seeking work), long commutes (% commuters, among those who commute to work alone, who drive longer than 30 min to work each day), fraction of population living in rural area, insufficient sleep (% reporting sleeping less than 7 h per night), fraction of population reporting excessive drinking, and fraction of traffic accident deaths with alcohol involvement.

This study was conducted in accordance with guidance from the Microsoft Ethics Review Board.

### Data availability

The data that support the findings of this study are available from Microsoft but restrictions apply to the availability of the data. Data are available from the authors on reasonable request and with permission of Microsoft.

## Electronic supplementary material

Supplementary Information

## Electronic supplementary material

**Supplementary information** accompanies the paper on the *npj Digital Medicine* website (10.1038/s41746-017-0003-3).
