# Supplementary material for: Psychomotor function measured via online activity predicts motor vehicle fatality risk
Source: NPJ Digit Med. 2018 Jan 15;1:20173. doi: 10.1038/s41746-017-0003-3 (PMC6550252; doi:10.1038/s41746-017-0003-3)
Supplement: Supplementary file 1 — Supplementary Information [file 41746_2017_3_MOESM1_ESM.docx]

Supplementary Information

Psychomotor Function Measured via Online Activity Predicts Motor Vehicle Fatality Risk

Tim Althoff, MS

Stanford University

althoff@cs.stanford.edu

Eric Horvitz, MD PhD

Microsoft

horvitz@microsoft.com

Ryen W. White, PhD

Microsoft

ryenw@microsoft.com

**Supplementary Figure 1:** Population-normalized average number of motor vehicle fatalities across US counties from 2009-2015. Fatality rates have been stable over many years supporting that the reported association between motor vehicle fatalities and keystroke timing based on 2016 data still hold.

**Supplementary Figure 2:** Year-to-year correlation between motor vehicle fatality rates of US counties (Average Pearson correlation: 0.895). This demonstrates that year-to-year changes have been small since 2009 and that using average mortality statistics of recent prior years is unlikely to influence our results. The main result we report is based on a strong correlation between keystroke timing and motor vehicle fatalities (Spearman ρ = 0.61; p « 10^−10^; N=2,723 counties), which could not be substantially affected by such variation.
